# Case Report: Acute superior mesenteric artery embolism combined with abdominal aortic aneurysm in an elderly female patient

**DOI:** 10.3389/fsurg.2025.1537980

**Published:** 2025-05-20

**Authors:** Ruixin Wu, Guofei Huang, Yang Zhou, Junwen He, Peiming Li

**Affiliations:** ^1^ School of Clinical Medicine, North Sichuan Medical College, Nanchong, China; ^2^ Department of Vascular Surgery, Deyang People’s Hospital, Deyang, China

**Keywords:** acute superior mesenteric artery embolism, abdominal aortic aneurysm, AngioJet mechanical thrombectomy, endovascular aneurysm repair, case report

## Abstract

**Background:**

Both acute superior mesenteric artery embolism (ASMAE) and abdominal aortic aneurysm (AAA) are insidious conditions that can lead to fatal outcomes. The coexistence of ASMAE and AAA in a single patient is rare.

**Methods and results:**

A 78-year-old female patient presented to our hospital due to abdominal pain for 10 h, with a diagnosis of AAA 2 h prior. Further evaluation through abdominal aorta computed tomography angiography (CTA) revealed the presence of both ASMAE and AAA. After a comprehensive assessment of her condition, treatment for ASMAE was prioritized. Digital subtraction angiography of the abdominal aorta and superior mesenteric artery (SMA) was performed, followed by local thrombolysis of the SMA embolism and two sessions of AngioJet mechanical thrombectomy. Once inflammation parameters have normalized and an active infection could be excluded, the patient subsequently underwent endovascular aneurysm repair (EVAR) for the AAA. Regular follow-up CTA over three years demonstrated that the SMA remained patent, and the abdominal aortic covered stent was intact, there were no significant endoleaks or thrombosis, with no evidence of stenosis in the abdominal aorta.

**Conclusion:**

The simultaneous occurrence of ASMAE and AAA is uncommon. ASMAE poses a significant threat to life and necessitates urgent treatment. Unruptured AAA can be treated electively once any contraindications have been addressed.

## Introduction

Acute superior mesenteric artery embolism (ASMAE) is a relatively rare but life-threatening disease, accounting for approximately 0.1% of hospitalized patients (1). Due to its insidious onset and inconsistent symptoms and signs, the clinical misdiagnosis rate is about 50%–60%, with a mortality rate that can reach 60%–80% (2). Therefore, timely diagnosis and treatment are of great significance for saving such patients. Abdominal aortic aneurysm (AAA) is also relatively uncommon in clinical settings, with an incidence of 0.5%–1% among elderly women over 65 years older (3). Similar to ASMAE, AAA has an insidious onset; however, once the aneurysm ruptures, the mortality rate can exceed 90% (4).

To the best of our knowledge, the simultaneous occurrence of ASMAE and AAA in a single patient is uncommon. Consequently, information regarding the diagnosis and treatment of these conditions is limited. In this report, we present a case involving an elderly female patient who suffers from both ASMAE and AAA.

## Case presentation

A 78-year-old female patient presented to our hospital due to abdominal pain. Ten hours ago, the patient suddenly experienced persistent, severe cramping pain in the upper abdomen without any obvious trigger, which was unbearable and accompanied by diarrhea three times. Two hours ago, a computed tomography (CT) scan of the abdomen at a local hospital indicated an AAA, leading to her transfer to our hospital for further treatment.

**Figure 1:**
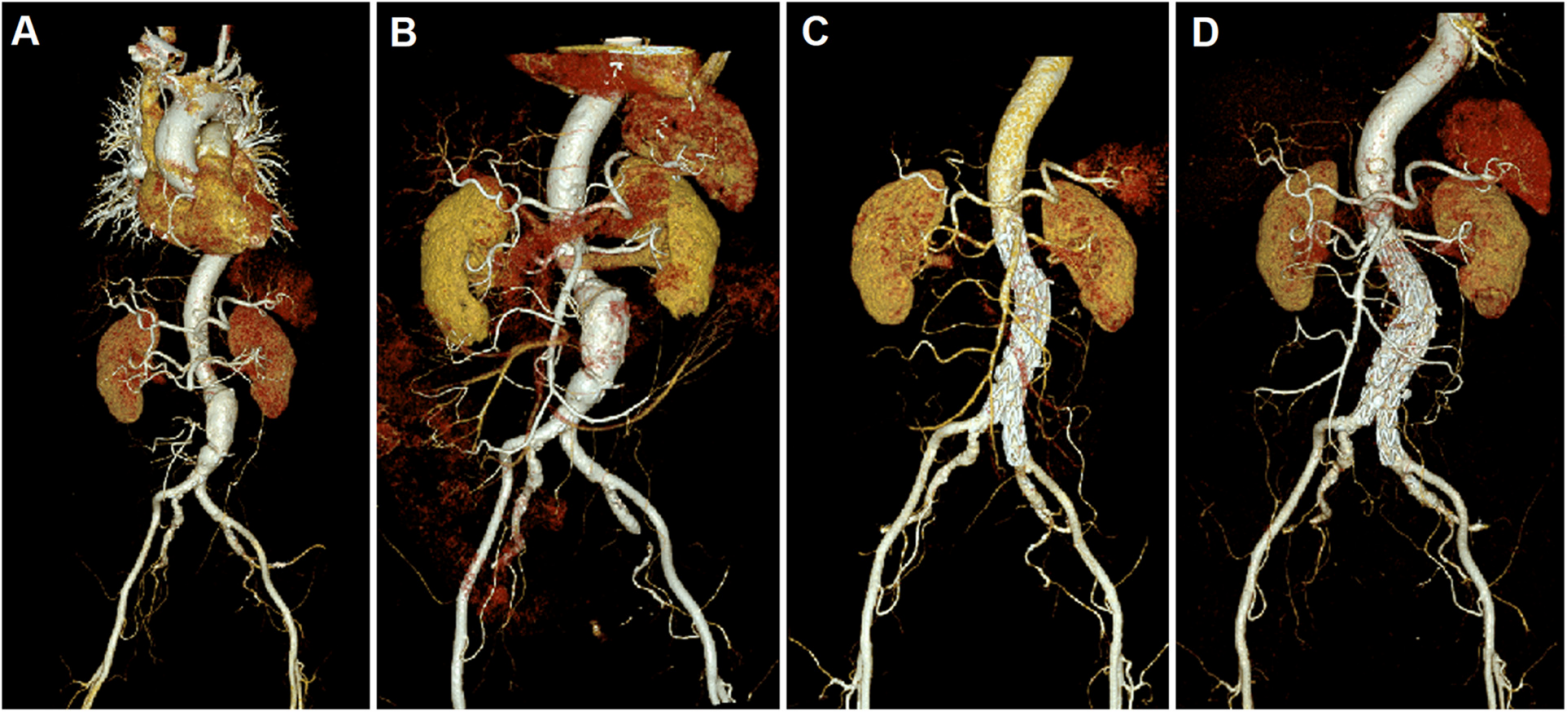
CTA figures before and after surgery. **(A)** Before surgery, an abdominal CTA indicated the presence of an ASMAE and an AAA. The larger cross-section of AAA was measured approximately 5.4 * 4.9 cm, with an involved length of about 8.8 cm; **(B)** A CTA performed on the 12th day after ASMAE treatment confirmed the patency of the main trunk and distal branches of the SMA; Follow-up CTA at 6 months **(C)** and 38 months **(D)** indicated that the SMA was patent, with no filling defects observed; the abdominal aortic covered stent was in place, with no significant endoleak or thrombosis.

The patient reported a 5-year history of hypertension, denied any history of heart disease, and has no other significant medical history. Upon admission, her blood pressure was recorded at 190/106 mmHg, accompanied by arrhythmia and a weak pulse. The abdominal examination indicated a soft abdomen with tenderness around the umbilicus, but no rebound tenderness or rigidity of the abdominal muscles. A pulsatile mass measuring approximately 5 × 5 cm was palpated in the abdomen, with a pulsation rhythm consistent with the heart rate. Auxiliary examinations revealed the following abnormities: white blood cell, 14.34 × 10^9^/L; absolute neutrophil count, 13.43 × 10^9^/L; neutrophil percentage, 93.7%; hypersensitive C-reactive protein, 44.41 mg/L, and serum sodium, 133.2 mmol/L. An electrocardiogram confirmed the presence of atrial fibrillation, and an abdominal CT angiography (CTA) indicated the presence of an ASMAE and an AAA (Figure 1A).

Considering the patient presented with both ASMAE and AAA, we initially attributed her abdominal pain to the ASMAE, but the possibility of impending rupture of the AAA could not be ruled out. Following a thorough assessment of her condition, treatment for the ASMAE was prioritized: digital subtraction angiography of the abdominal aorta and superior mesenteric artery (SMA) was performed, followed by local thrombolysis of the SMA embolism (200,000 units of urokinase were administered directly onto the embolism for 30 min) and two sessions of AngioJet mechanical thrombectomy. Post-procedure, the patient experienced a significant reduction in abdominal pain, which had largely resolved within 12 h. Since infection could not be proved, the patient was prophylactically treated with Cefoperazone/tazobactam sodium (2 g, ivgtt, q8 h) to prevent bacteria translocation. Additionally, enoxaparin sodium was administered (5,000 IU, sc, q12) for anticoagulation. 12 days later, the patient's inflammatory markers returned to normal, and her abdominal pain completely subsided, with no signs of tenderness, rebound tenderness, or abdominal muscle rigidity. A CTA performed on the 12th day post-procedure confirmed the patency of the main trunk and distal branches of the SMA (Figure 1B). Subsequently, an endovascular aneurysm repair (EVAR) procedure was performed to address the AAA: bilateral femoral arteries were incised, followed by abdominal aorta angiography, endovascular stent-graft exclusion, bilateral femoral artery endarterectomy, and angioplasty. The patient recovered well post-surgery, and a follow-up abdominal aorta CTA indicated patency of the SMA with no significant filling defects; the covered stent in the abdominal aorta was patent with no evidence of endoleak or stenosis. The patient was discharged at the 24th day after admission.

After discharge, the patient was prescribed long-term oral dabigatran (110 mg, bid) for anticoagulation and atorvastatin (10 mg, qd) for lipid regulation. Follow-up CTA at 6 months (Figure 1C) and 38 months post-procedure (Figure 1D) indicated that the SMA was patent, with no filling defects observed; the abdominal aortic covered stent was in place, with no significant endoleak or thrombosis, and there was no evidence of stenosis in the abdominal aorta.

## Discussion

CTA is a valuable tool for the diagnosis of both ASMAE and AAA. The patient in this case presented to our hospital due to abdominal pain, initially suspected to be related with an impending ruptured or a ruptured AAA, while ASMAE was not considered at first. Upon further evaluation, the physician noted that the patient had atrial fibrillation, and the presenting symptoms and signs were not sufficient to fully support the diagnosis of an impending ruptured or a ruptured AAA. At that time, the patient's vital signs were stable, and an urgent abdominal aortic CTA was performed, which ultimately revealed the presence of both ASMAE and AAA. The simultaneous occurrence of these two conditions in a single patient is rare, underscoring the importance of timely CTA for accurate diagnosis in such cases.

The management of ASMAE should follow the 4R treatment strategy: resuscitation, rapid diagnosis, revascularization, and reassessment of bowel (5). Once the diagnosis is confirmed, timely intervention is essential for the patient's prognosis. Currently, the primary treatment modalities for ASMAE include traditional open surgeries and endovascular interventions. Surgical procedures, including thrombectomy, endarterectomy, and bypass surgery, have the advantage of effectively removing thrombus and clearly determining whether there is intestinal necrosis, allowing for the resection of the affected intestinal segment if necessary (6). However, ASMAE is frequently observed in elderly patients with poor nutritional status and multiple chronic comorbidities. For these patients, open surgeries tend to be highly invasive and associated with a higher risk of complications, making them less appropriate as a treatment choice. Endovascular interventions encompass techniques such as catheter-directed thrombolysis, mechanical thrombectomy (such as Rotarex, AngioJet and Penumbra thrombectomies), balloon angioplasty, and stent placement. Over the past decade, an increasing number of reports have successfully demonstrated the value and advantages of these techniques in the treatment of ASMAE (7–11). According to the literature, if a patient is in the early stage of ASMAE (within onset of a disease less than 12 h), shows no signs of peritonitis or intestinal necrosis, and has a suspected or confirmed embolus on CTA, endovascular thrombectomy with or without thrombolysis can be attempted (12). In this case, the elderly patient presents with poor cardiopulmonary function, preoperative hypertension and an AAA, rendering her unsuitable for extensive open surgery. The AngioJet mechanical thrombectomy is widely used for the removal of deep vein thrombosis in the lower limbs (13). In the context of ASMAE, studies have also demonstrated its safety and efficacy. For example, Yu et al. conducted a retrospective analysis involving 12 patients with ASMAE who underwent percutaneous mechanical thrombectomy (PMT). Among these patients, 6 were treated solely with the AngioJet device, while the other 6 received a combination of the AngioJet device and catheter thrombectomy. The results indicated that 10 patients achieved complete thrombus removal, while 2 experienced partial removal. Importantly, no complications related to PMT were reported, except for one patient who developed a postoperative pseudoaneurysm (14). In this case, ASMAE was successfully managed using local thrombolysis and two sessions of AngioJet thrombectomy.

Most patients with AAA experience gradual enlargement of the aneurysm, and even small aneurysms can have a risk of rapid growth and rupture. Factors such as the aneurysm diameter, hypertension and female gender are associated with aneurysm rupture. International guidelines recommend that women with an aneurysm diameter greater than 5 cm (for man, greater than 5.5 cm) should be considered for elective surgery (15), while Chinese guidelines suggest that women with a diameter greater than 4.5 cm (for man, greater than 5.0 cm) may be considered for elective surgery (16). In this case, the female patient has a history of hypertension and atrial fibrillation. Upon admission, the CTA indicated that the aneurysm diameter was approximately 5.4 cm. During the surgical treatment for the ASMAE, imaging revealed an aneurysmal dilation of the abdominal aorta, with the widest part measuring about 5.2 cm and a length of approximately 8 cm, indicating surgical intervention was warranted. However, at that time, the patient had elevated inflammatory markers, and uncontrolled active infection is a contraindication for the treatment of unruptured AAA (16). To mitigate the risk of aneurysm rupture, we decided to proceed with AAA treatment only after successfully controlling the infection. The treatment for AAA primarily includes open surgery and EVAR. Open surgery is suitable for AAA patients who are in good overall health and have controllable surgical risks (17). Conversely, EVAR is a minimally invasive procedure that significantly reduces surgical trauma and shortens hospital stays, making it especially suitable for patients with compromised cardiopulmonary function and other high-risk factors (17–19). Consequently, our patient's AAA was successfully treated using EVAR.

## Conclusion

The simultaneous occurrence of ASMAE and AAA is uncommon. ASMAE poses a significant threat to life and necessitates urgent treatment. Unruptured AAA can be treated electively once any contraindications have been addressed.

## Data Availability

The original contributions presented in the study are included in the article/Supplementary Material, further inquiries can be directed to the corresponding author.
